# Increased care-need in older long-term care insurance users after the 2018 Japan Floods: a retrospective cohort study based on the Japanese long-term care insurance claims

**DOI:** 10.1265/ehpm.22-00269

**Published:** 2023-05-17

**Authors:** Kotaro Ikeda, Shuhei Yoshida, Yuji Okazaki, Daisuke Miyamori, Saori Kashima, Shinya Ishii, Soichi Koike, Keishi Kanno, Masanori Ito, Masatoshi Matsumoto

**Affiliations:** 1 Department of General Internal Medicine, Hiroshima University Hospital, Hiroshima, Japan; 2 Department of Community-Based Medical System, Graduate School of Biomedical and Health Sciences, Hiroshima University, Hiroshima, Japan; 3 Department of Emergency Medicine, Hiroshima City Hiroshima Citizens Hospital, Hiroshima, Japan; 4 Environmental Health Sciences Laboratory, Graduate School of Advanced Science and Engineering, Hiroshima University, Hiroshima, Japan; 5 Center for the Planetary Health and Innovation Science, The IDEC Institute, Hiroshima University, Hiroshima, Japan; 6 Department of Medicine for Integrated Approach to Social Inclusion, Graduate School of Biomedical and Health Sciences, Hiroshima University, Hiroshima, Japan; 7 Division of Health Policy and Management, Center for Community Medicine, Jichi Medical University, Tochigi, Japan

**Keywords:** Long-term care insurance, Activities of daily living, Natural disasters, The 2018 Japan Floods, Rural health services

## Abstract

**Background:**

Level of care-need (LOC) is an indicator of elderly person’s disability level and is officially used to determine the care services provided in Japan’s long-term care insurance (LTCI) system. The 2018 Japan Floods, which struck western Japan in July 2018, were the country’s second largest water disaster. This study determined the extent to which the disaster affected the LOC of victims and compared it with that of non-victims.

**Methods:**

This is a retrospective cohort study, based on the Japanese long-term care insurance claims from two months before (May 2018) to five months after the disaster (December 2018) in Hiroshima, Okayama, and Ehime prefectures, which were the most severely damaged areas in the country. A code indicating victim status, certified by a residential municipality, was used to distinguish between victims and non-victims. Those aged 64 years or younger, those who had the most severe LOC before the disaster, and those whose LOC increased even before the disaster were excluded. The primary endpoint was the augmentation of pre-disaster LOC after the disaster, which was evaluated using the survival time analysis. Age, gender, and type of care service were used as covariates.

**Results:**

Of the total 193,723 participants, 1,407 (0.7%) were certified disaster victims. Five months after the disaster, 135 (9.6%) of victims and 14,817 (7.7%) of non-victims experienced the rise of LOC. The victim group was significantly more likely to experience an augmentation of LOC than the non-victim group (adjusted hazard ratio 1.24; 95% confidence interval 1.06–1.45).

**Conclusions:**

Older people who were affected by the disaster needed more care than before and the degree of care-need increase was substantially more than non-victims. The result suggests that natural disasters generate more demand for care services among the older people, and incur more resources and cost for society than before.

**Supplementary information:**

The online version contains supplementary material available at https://doi.org/10.1265/ehpm.22-00269.

## Introduction

In recent years, the frequency and intensity of natural disasters have increased worldwide [1]. With the aging of the population worldwide [2], it is becoming increasingly important to understand the health-related effects of disasters, especially on older people, who are particularly vulnerable to disasters. Furthermore, with the number of people requiring nursing care increasing in recent years [3], there is an increasing need for scientific evidence to show the impact of natural disasters on the quality of life of older people requiring nursing care.

In Japan the level of disability, which is employed in the public nursing care insurance, is represented as “level of care-need (LOC)”. LOC is determined based on various bio-psycho-social factors such as activities of daily living (ADL), cognitive function, and the ability to participate in social activities such as accounting and shopping. Each elderly person has a certified LOC, and based on the LOC, receives certain type and amount of care services in the long-term care insurance (LTCI) system which covers the entire elderly population of Japan. Knowing the potential change in LOC caused by natural disasters helps health and welfare professionals as well as policy makers, respond quickly and appropriately to the change of care-need of the victims.

Older residents affected by natural disasters may experience a decline in activities of daily living (ADL) [4]. Natural disasters have been known to cause various diseases [5]. In particular, trauma and cerebrovascular diseases are considered to worsen ADL of older people after natural disasters. Psychiatric disorders such as depression, depressive states, and post-traumatic stress disorder (PTSD), which can also be caused by natural disasters, would deteriorate ADL [6]. Furthermore, the physical activity of older people seems to decrease due to emergency hospitalization or living in an unusual environment such as a refugee shelter [7, 8]. Cognitive function is also reportedly impaired by natural disasters [9, 10]. These illnesses can occur not only in older victims themselves but also in family members who provide daily care to the victims. Damage to the physical and community environment such as destruction of the residence and tearing apart from neighbors also influences the care for older people. Therefore, it is rational to consider that natural disasters potentially increase the LOC of older victims although the relationship has not been studied so far.

From June 28 to July 8, 2018, heavy torrential rains took place over a wide area of western Japan, which was termed “the 2018 Japan Floods.” The Floods broke the record for the amount of rainfall in many areas of Japan and caused devastating damage. Approximately 28,000 residents were evacuated across Japan, with 237 dead, 8 missing, and 432 injured. Many houses were inundated; 6,767 were completely destroyed, 15,234 were partially destroyed or damaged, and 28,467 were flooded [11]. This torrential rain caused economic damage of approximately 1.2 trillion yen (USD 9.2 billion) throughout Japan [12], making it the second largest water disaster in history. Damage was particularly concentrated in Hiroshima, Okayama, and Ehime prefectures, with these three prefectures alone accounting for 89% of all deaths and 78% of all house damage [13].

The purpose of this study is to investigate whether the 2018 Japan Floods caused a rise in the LOC of older people who were using LTCI services. It was hypothesized that the natural disaster has worsened the LOC of older people and increased the use of care services due to decline in ADL, impaired cognitive function, loss of family members, and destruction of the physical and community environment. The Japanese long-term care insurance claims contained the precise LOC data of all the residents of these prefectures who used LTCI services within the period. It also included information on who were certified by local governments as direct victims of the 2018 Japan Floods. The database was thus used to examine the change in the LOC before and after the natural disaster and measure the impact of the disaster on the LOC of older disaster victims as compared to non-victims.

## Methods

### Study design, settings, and data collection

This is a longitudinal, retrospective cohort study observing LTCI users between May 2018 and December 2018. The Japanese long-term care insurance claims was used to conduct this study. Three prefectures (Hiroshima, Okayama, and Ehime), which were the hardest hit areas by the disaster, were selected as the study area, and residents who had used LTCI for at least two months in the pre-disaster period (from May to July 2018) were included as study participants. The study participants were limited to those who were 65 years old or older. Although LTCI in Japan is available to all citizens aged 40 and older, for those aged between 40 and 64 years, LTCI is available only if they are afflicted by a limited group of diseases. Therefore, this study includes only those aged 65 and older, who are certified regardless of the cause. To avoid observing LOC changes not related to the disaster, those whose LOC had already changed in the pre-disaster period and those who newly started to use LTCI services after the disaster were excluded. Residents whose baseline LOC was Care Level 5, which was the highest level of care-need and cannot increase further, were also excluded. Information on age group, gender, disaster-victim status, baseline LOC, and care services used by each participant was extracted from the database. Age was presented as seven groups of five years starting at 65 years, with the oldest group being 95 years and older.

### Description of the levels of care-need

Of the 36.19 million older people in Japan, more than 6.8 million receive LTCI services. Users of LTCI services are graded according to their officially certified LOC, and the type and amount of care services covered by the insurance are determined according to the LOC [14, 15]. There are seven grades of LOC, graded in descending order from mildest to most severe: Support Level 1, Support Level 2, and Care Level 1 to 5. Variables such as physical condition of the living house, having a chronic disease, degree of independence in daily living, presence of dementia and mental illness, physical condition including muscle strength and presence of pressure ulcers, and predictions of possible future disabilities are measured by the attending physician for each older individual. The provisional LOC is determined mainly from the estimated time needed to provide care for the person calculated by a computer program based on the results of a primary check-up by an official of the residential municipality regarding the person’s health and socioeconomic status. Based on the result, a specialized committee of the local government, including physicians and other health professionals, determines the final LOC. In addition to these factors, the availability of family care-givers and other family condition are also examined, although they are not a part of the criteria for certification.

In particular, physical function, mental status, and cognitive function are recognized as important factors in the decision of LOC. The Support Levels are a state in which a person is able to live on their own but needs partial assistance. The Care Levels indicate a state in assistance is required on a daily basis due to a cognitive impairment and/or a decline in motor skills. Especially, Care Level 5 indicates severe conditions such as a complete bedridden state. The higher the LOC, the greater the range and amount of care services that can be used under the LTCI coverage. The co-payment for the use of LTCI services ranges from 10% to 30% of the service fee, depending on the income level of the user of LTCI services.

LTCI services include home-visit services provided by nurses or rehabilitators, day services provided during the daytime by rehabilitation professionals in facilities, short-stay services for respite care in facilities, in-facility services providing long-term residence care, multifunctional services that provide home-visit services, day services, and short-stay services in one place, and rental services of welfare equipment. Generally, the day service and short-stay facilities are used when the LOC is lower (such as Care Level 1 and 2), whereas the in-facility services are used when the LOC is higher (Care Level 4 and 5) [16]. The certified LOC is valid for 6 and 12 months for a new and repeated certification, respectively. A change of LOC classification can be applied for before the next expiration date of the current certification if the condition of the person changes. This type of change of LOC is called “category change”.

### Definition of disaster victim

The Japanese long-term care insurance claims used in this study contained a “disaster-victim code” which showed whether a person was certified as a disaster victim by a municipality. Therefore, it was possible to categorized each individual in the database into victim and non-victim groups using those code. An individual who met any of the following conditions was certified as a disaster victim: the house was completely or partially destroyed, burned down, flooded above floor level, or suffered similar damage; the primary breadwinner died or suffered serious injury or illness; the primary breadwinner’s whereabouts were unknown; the primary breadwinner ceased or was suspended to work; and the primary breadwinner lost their job and currently has no income. A person who was certified as a disaster victim was exempted from the co-payment for the use of LTCI services throughout the observation period.

### Outcomes

To evaluate the augmentation of LOC due to the 2018 Japan Floods, the primary outcome was defined as the augmentation of LOC during the observation period. The occurrence of an outcome was defined as a rise of at least one grade of LOC from the pre-disaster (May–July 2018) to post-disaster (August–December 2018) period. Once the LOC increased, another change was not considered. This outcome was presented as a cumulative incidence rate for the victim and non-victim groups. The observation was terminated at the end of December 2018.

### Statistical analysis

The data presented in the baseline characteristics were categorical variables, summed by proportion. Each value was compared between the victims and non-victims by performing the chi-squared test. There were no missing data in this database.

In the main analysis, the cumulative incidence of LOC augmentation was calculated using the Kaplan-Meier method, and inter-group differences in incidence were compared by performing the log-rank test. Furthermore, the hazard ratio (HR) of the victim group to the non-victim one was calculated. The HRs were reported with 95% confidence intervals. The model was adjusted for age group, sex, LOC before the disaster, and care services used before the disaster. Use of each care service was treated as a dichotomous variable in which use was 1 and non-use was 0. Cluster-robust standard errors were used for adjusting for municipalities where the individuals lived. The values were reported with 95% confidence intervals. Age, sex, differences in care services used, cognitive function, and underlying medical conditions are known risk factors for changes in LOC [17–19]. Therefore, multivariate analysis was performed using four of these items as covariates: age, sex, baseline LOC, and care services used. In addition, logistic regression analysis was performed to evaluate the robustness of this model using the same outcome and covariates. Furthermore, multilevel logistic regression analysis was performed to adjust for municipalities where the individuals lived.

Subgroup analysis of each age, sex and care service group was also performed to examine the heterogeneity of the participants. The primary outcome was evaluated in each of the three subgroups: age, sex, and baseline LOC. For the age group, age 80 was used as a delimiter, which is the middle value in the present data set. The Cox proportional hazards model was used in the analysis to calculate the HR of the victim to the non-victim group adjusted for the four covariates. An interaction term test was also performed to assess the interaction between disaster suffering and each of the subgroups. All statistical analyses ware performed using Stata/MP version 16 (Stata Corp, 2019), and a P value < 0.05 (two-tailed) was considered statistically significant. For the interaction term test, a P value < 0.1 (two-tailed) was considered statistically significant.

## Results

**Fig. 1:**
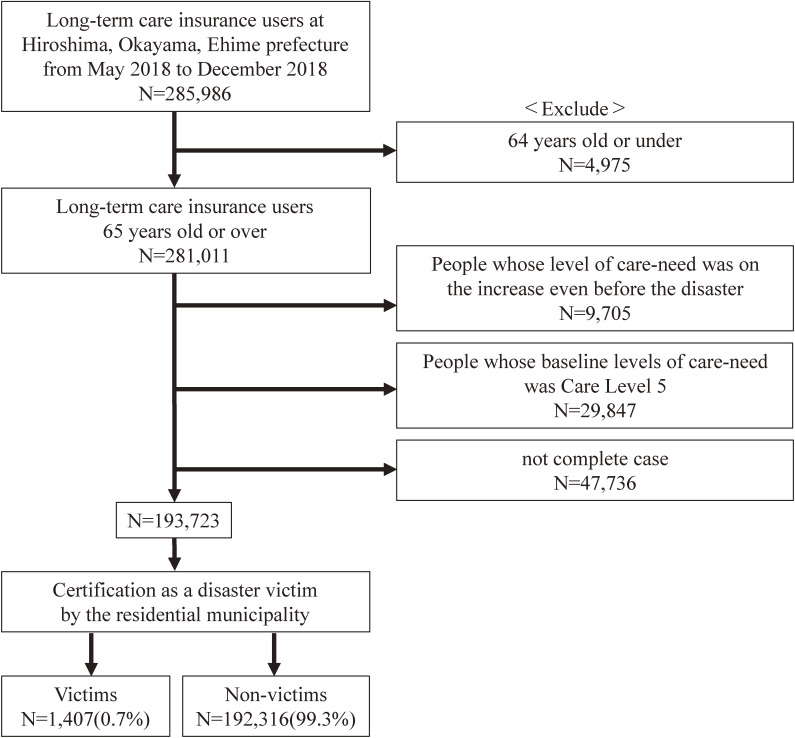
Flow diagram for the selection of study participants.

There were 285,986 people and 292 municipalities in the database used in this study. Of those individuals, 4,975 people were 64 years old or younger, 9,705 people who experienced an increase of LOC even before the disaster (from May to July 2018), and 29,847 residents whose LOC was Care Level 5 before the disaster. These individuals were excluded. Consequently, 193,723 people were included in the final analysis. The victims and non-victims consisted of 1,407 (0.7%) and 192,316 (99.3%) participants, respectively (Fig. 1).

Table 1 shows the baseline characteristics of the participants. There was no statistically significant difference between victims and non-victims in terms of age and sex. The victims consisted of 1,142 (81.2%) persons requiring nursing care (Care Level 1–4), and the non-victims consisted of 153,089 (79.6%) persons requiring nursing care. Before the disaster, the utilization of home visit and facility services was less frequent, and the utilization of day and rental services was more frequent among the victims than the non-victims. The number of residents who did and did not experience an increase in LOC was 17,748 (9.15%) and 175,995 (90.85%), respectively.

**Table 1:** Baseline characteristics of the study participants.

	**Total**	**Victims**	**Non-victims**	***P* value^†^**	**Increase ** **of LOC^‡^**	**Non-increase ** **of LOC^‡^**	***P* value^†^**
	**(N = 193,723)**	**(0.7%)**	**(99.3%)**		**(9.15%)**	**(90.85%)**	
		**(N = 1,407)**	**(N = 192,316)**		**(N = 17,728)**	**(N = 175,995)**	
**Age, categorical (%)**				0.13			<0.001
65–69 years old	7,295 (3.8)	49 (3.5)	7,246 (3.8)		501 (2.8)	6,794 (3.9)	
70–74 years old	12,423 (6.4)	77 (5.5)	12,346 (6.4)		948 (5.4)	11,475 (6.5)	
75–79 years old	22,174 (11.5)	171 (12.2)	22,003 (11.4)		1,753 (9.9)	20,421 (11.6)	
80–84 years old	42,102 (21.7)	274 (19.5)	41,828 (21.8)		3,578 (20.2)	38,524 (21.9)	
85–90 years old	55,720 (28.8)	438 (31.1)	55,282 (28.8)		5,147 (29.0)	50,573 (28.7)	
90–94 years old	38,834 (20.1)	278 (19.8)	38,556 (20.1)		4,007 (22.6)	34,827 (19.8)	
95 years old <	15,175 (7.8)	120 (8.5)	15,055 (7.8)		1,794 (10.1)	13,381 (7.6)	
**Sex**							
Men, n (%)	53,639 (27.7)	411 (29.2)	53,228 (27.7)	0.2	4,897 (27.6)	48,742 (27.7)	0.8
**Baseline levels of care-need (%)**				0.17			<0.001
Support Level 1	16,944 (8.8)	98 (7.0)	16,846 (8.8)		1,749 (9.9)	15,195 (8.6)	
Support Level 2	22,548 (11.6)	167 (11.9)	22,381 (11.6)		1,577 (8.9)	20,971 (11.9)	
Care Level 1	49,506 (25.6)	375 (26.7)	49,131 (25.6)		5,435 (30.7)	44,071 (25.0)	
Care Level 2	43,171 (22.3)	323 (23.0)	42,848 (22.3)		3,875 (21.9)	39,296 (22.3)	
Care Level 3	33,210 (17.1)	226 (16.1)	32,984 (17.2)		3,102 (17.5)	30,108 (17.1)	
Care Level 4	28,344 (14.6)	218 (15.5)	28,126 (14.6)		1,990 (11.2)	26,354 (15.0)	
Type of LTCI* services in use (%)							
Home-visit services	48,631 (25.1)	339 (24.1)	48,292 (25.1)	0.38	4,195 (23.7)	44,436 (25.3)	<0.001
Day services	94,248 (48.7)	767 (54.4)	93,481 (48.6)	<0.001	8,001 (45.1)	86,247 (49.0)	<0.001
Short-stay services	18,944 (9.8)	162 (11.5)	18,782 (9.8)	0.03	2,184 (12.3)	16,760 (9.5)	<0.001
In-facility services	50,751 (26.2)	339 (24.1)	50,412 (26.2)	0.07	5,784 (32.6)	44,967 (25.6)	<0.001
Multifunctional facility	1,488 (0.77)	14 (1.0)	1,474 (0.8)	0.33	178 (1.0)	1,310 (0.7)	<0.001
Rental services of equipment	94,078 (48.6)	680 (48.3)	93,398 (48.6)	0.86	7,804 (44.0)	86,274 (49.0)	<0.001

*LTCI, long-term care insurance; †, chi-squared test; ‡LOC, levels of care-need

**Fig. 2:**
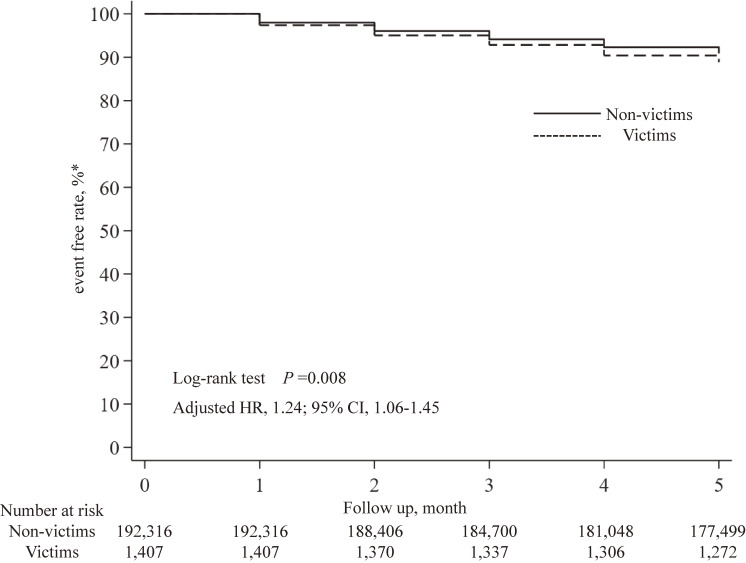
Kaplan-Meier curves of victims and non-victims whose care-need level did not increase after the disaster. Month 0 (July 2018) indicates the month when the 2018 Japan Floods occurred. HR, Hazard Ratios; CI, confidence interval. HR was adjusted for age, sex, baseline LOC, care services used before the disaster, and municipalities where the individuals lived. * An event was counted when a participant’s care-need level increased at any moment within the post-disaster period.

Figure 2 shows the cumulative event-free rate of LOC-rise in the victims and non-victims during the five months after the disaster. The augmentation of LOC occurred in 9.6% of the victims (N = 135) and 7.7% of the non-victims (N = 14,817) during the five months, indicating that LOC had a greater statistically significant effect among victims than non-victims (adjusted HR 1.24; 95% confidence interval 1.06–1.45) (Table 2). In addition, logistic regression analysis showed that the LOC of older victims tended to increase after the disaster (adjusted Odds Ratio 1.29; 95% confidence interval 1.08–1.52) (Supplementary Table 1).

**Table 2:** Adjusted hazard ratios of an increase in the level of care-need.

	**Adjusted HR^†^**	***P* value**	**[95% CI^‡^]**	
**Victims**	1.24	0.005	1.06	1.45
**Age categorical**				
65–69 years old	Ref	Ref	Ref	Ref
70–74 years old	1.10	0.019	1.00	1.22
75–79 years old	1.12	<0.001	1.01	1.25
80–84 years old	1.19	<0.001	1.09	1.30
85–90 years old	1.28	<0.001	1.16	1.40
90–94 years old	1.42	<0.001	1.30	1.55
95 years old <	1.66	<0.001	1.50	1.83
**Sex**				
Women	Ref	Ref	Ref	Ref
Men, n (%)	0.92	<0.001	0.89	0.96
**Baseline levels of care-need (%)**				
Support Level 1	Ref	Ref	Ref	Ref
Support Level 2	0.66	<0.001	0.59	0.74
Care Level 1	0.93	0.31	0.81	1.07
Care Level 2	0.69	<0.001	0.57	0.84
Care Level 3	0.62	<0.001	0.51	0.75
Care Level 4	0.42	<0.001	0.35	0.52
Type of LTCI* services in use**				
Home-visit services	1.12	<0.001	1.08	1.17
Day services	0.996	0.85	0.96	1.04
Short-stay services	1.57	<0.001	1.50	1.63
In-facility services	1.83	<0.001	1.71	1.95
Multifunctional facility	1.59	<0.001	1.34	1.88
Rental services of equipment	1.05	0.03	1.00	1.10

*LTCI, long-term care insurance; †, hazard ratio; ‡, CI, confidence interval; ** In each service, those who did not use the service were used as reference. HR was adjusted for age, sex, baseline LOC, care services used before the disaster, and municipalities where the individuals lived.

**Fig. 3:**
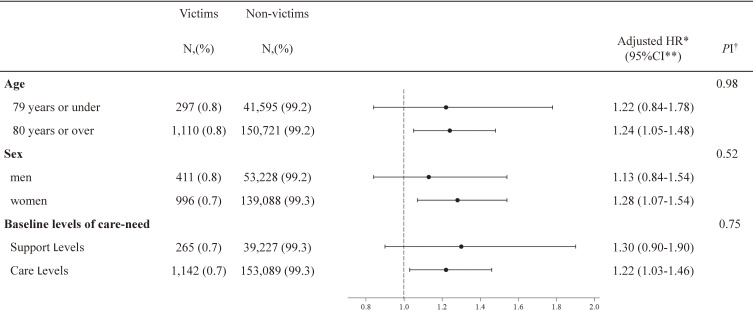
Forest Plot of Hazard Ratios for augmentation of level of care-need: A subgroup analysis. *HR, Hazard Ratios; HR was adjusted for age, sex, baseline LOC, care services used before the disaster, and municipalities where the individuals lived.; **CI, confidence interval; †PI, P for Interaction.

Figure 3 shows the results of the subgroup analysis. In the subgroup divided by age, the adjusted HR was 1.22 (95% confidence interval 0.84–1.78) and 1.24 (95% confidence interval 1.05–1.48) for the group aged 79 years or younger and aged 80 years or older, respectively. There was no interaction between age and disaster (P = 0.98). In the subgroup divided by sex, the adjusted HR was 1.13 (95% confidence interval 0.84–1.54) and 1.28 (95% confidence interval 1.07–1.54) for men and women, respectively. There was no interaction between sex and disaster (P = 0.52). In the subgroup divided by baseline LOC, the adjusted HR was 1.30 (95% confidence interval 0.90–1.90), and 1.22 (95% confidence interval 1.03–1.46) for the Support Level and Care Level groups, respectively. Furthermore, an interaction term test between baseline LOC and disaster showed no statistically significant difference (P = 0.75). Therefore, no heterogeneity was identified in any of the subgroups.

## Discussion

This study showed that older LTCI users affected by the 2018 Japan Floods were more likely to suffer an increase in care-needs than those who were not affected. The effect was observed throughout the five months after the disaster. To the best of our knowledge this is the first report showing the association between a natural disaster and the care-need of older people. The results suggest natural disasters cause physical, psychological, and environmental damage to older people and impose a heavy burden on society as well as on older people themselves.

Studies conducted during the 2011 Great East Japan Earthquake, the largest natural disaster in recorded history, have suggested that the rate of newly certified LTCI users increased among those who lived in the affected areas, and revealed an increasing trend of caregiving costs among those who evacuated [20–22]. However, these reports were not based on data of each disaster victim but the population-level data of the affected area as a whole. Moreover, they evaluated surrogate outcomes of LOC rise, such as a change in LTCI certification rate and LTCI cost among the whole population of the area. Population-level statistics do not necessarily reflect what happens in each individual (ecological fallacy). To overcome these problems existing in the previous reports, this study used an all-inclusive individual-level database of the affected region that also included the disaster status of each participant authorized by a local government. This database made it possible to directly assess the augmentation of LOC of each victim compared to that of each non-victim. Thus, the results suggested more clearly the impact of a natural disaster on the care-need level of older residents.

Factors influencing the LOC include physical (such as femur fracture, vertebral fracture and stroke) [23–25], psychological (such as depression and cognitive decline) [26], and social conditions [27–29]. The exacerbation of these factors would have a strong influence on the augmentation of care-need. Victims of a natural disaster often suffer from a combination of these three factors. For example, the incidence of cerebral infarction increases by more than twice among disaster-affected older men than non-affected ones [30]. Cerebral hemorrhage has also been reported to increase after a natural disaster [31]. Furthermore, it has been shown that the cognitive function of older victims of the 2018 Japan Floods has been impaired more than that of non-victim older residents [9, 10]. In addition, natural disasters can worsen social frailty due to the destruction of homes and the loss of care givers and friends, which may lead to physical and psychological deterioration. Therefore, victims are supposedly affected by these physical, psychological, and social factors, which may lead to the augmentation of LOC.

It is noteworthy that in the present study, the LOC in the victims increased within the five-month period after the disaster. This means that the impact of natural disaster on the need for long-term care can appear immediately after the disaster. The immediate increase in medical need after a disaster is well known [32, 33]; this study added evidence that a similar phenomenon takes place for long-term care. In the immediate aftermath of disaster, problems unique to this period can occur, such as the need to provide nursing care beds and other nursing care items at evacuation shelters, and the need to move care-takers to nearby non-affected nursing care facilities. Furthermore, the results of this study indicate that the LOC of older victims even in a post-acute phase, which is a few months after the disaster, progressively increases, resulting in a stepwise increase in the demand for nursing care during the post-disaster period. This suggests that policy makers and LTC providers are required to anticipate and prepare for not only the acute phase expansion of care need but also the constant and long-term increase of care-need among victims though year-long observation was not possible in this study.

In the subgroup analyses, the hazard ratios for increasing LOC after the disaster were not statistically significant in the groups younger than 80 years, men, and those in Support Levels as baseline LOC. Furthermore, the interaction terms were not statistically significant among all groups, and the point estimates were almost the same in each paired group. This can be due to the limited statistical power derived from the small number of events in each group.

This study has some limitations. First, the people in need of care identified in this study are people “certified as needing nursing care,” and not all people actually in need of nursing care. The database includes only those who are certified as needing support or have disabilities. Therefore, it is not possible to measure how older people who did not use the LTCI services at all before the disaster became in need of them after the disaster.

Second, this database does not include data on diseases. Since there is no information on the underlying diseases or comorbidities that led to the certification of LOC, it is impossible to ascertain whether a new disease or an exacerbation of the underlying disease was the cause of the rise of LOC. In Japan, connecting the personal IDs of the national database and that of the Japanese long-term care insurance claims was started in October 2020 [34], which is after the provision of the data for this study. Therefore, the data could not be matched in this study. When the connected database is widely available, we can further examine the medical causes of the disaster-derived increase in LOC, and to prevent the increase in LOC by early medical intervention to the high risk people.

Third, information on death is not included in the database. Therefore, the observation of participants who were dead during the observation period could not be terminated. When a person dies, the whole LCTI service is discontinued since that moment in the database. Although we did observe all cases of LOC rise, those who discontinued the services, that is those who potentially died, were not included. The victim group would have had a higher mortality rate than the non-victim group during the observation period, and if the service termination case due to death were included, the difference in results between victims and non-victims would have been more pronounced.

Fourth, the percentage of residents who recently started using LTCI to the total population was unknown because non-users of LTCI services before the disaster were not included in this database. Without non-user information, the proportion of cases with a new LOC certification among all the will-be-victims could not be calculated because there were no data on those who had not used LTCI services at all during the period. If new certification and the initiation of LTCI use were counted as an augmentation of LOC, the results might have changed.

Fifth, several factors which can be related to the augmentation of LOC such as socioeconomic status, the number of family caregivers, and whether the change of LOC was “category change” or not, remained unadjusted, because these factors could not be used in this database. In addition, with this database, the year-long effect of the disaster could not be evaluated because of the limitation of the study period. Due to the nature of the care level certification, a change in general condition may be reflected in the LOC with a delay of about several months. On the other hand, the victims were exempted from the co-payment for LTCI use, and it is possible that the deterioration of their general condition was picked up earlier in the victims than the non-victims. These issues should be addressed in future research.

In recent years, the frequency and intensity of natural disasters have been increasing worldwide, and in addition, the population is aging. The issue of how to protect vulnerable people and reduce economic losses due to natural disasters is an important issue worldwide [35]. Although various disaster preparedness measures for the vulnerable older residents have been proposed [36–38], the preparedness for post-disaster care is largely ignored. New guidelines for the care of older residents during and after disasters incorporating the results of this study are needed.

## Conclusion

This study showed that within five months after the event of a natural disaster, older victims were more likely to suffer a rise in care-need than older non-victims. The result suggests that natural disasters generate more demand for care services among older people. It also means there was an increased burden on family members and higher social security costs for society. Acknowledgement of this fact and preparedness for the expected outcome are needed for health and welfare professionals and policy makers.

## Abbreviations

LOC: level of care need
LTCI: long-term care insurance
ADL: activities of daily living
PTSD: post-traumatic stress disorder
HR: hazard ratio

## Supplementary information

**Additional file 1:** **Supplementary Table 1.** Adjusted odds ratios of an increase in the level of care-need.

## Declarations

### Availability of data and materials

The data that support the findings of this study are not publicly available due to restrictions stipulated by the Ministry of Health, Labour and Welfare.
